# Malnutrition, Hypertension Risk, and Correlates: An Analysis of the 2014 Ghana Demographic and Health Survey Data for 15–19 Years Adolescent Boys and Girls

**DOI:** 10.3390/nu12092737

**Published:** 2020-09-08

**Authors:** Fusta Azupogo, Abdul-Razak Abizari, Elisabetta Aurino, Aulo Gelli, Saskia J. M. Osendarp, Hilde Bras, Edith J. M Feskens, Inge D. Brouwer

**Affiliations:** 1 Division of Human Nutrition and Health, Wageningen University and Research, 6708 PB Wageningen, The Netherlands; saskia.osendarp@wur.nl (S.J.M.O.); edith.feskens@wur.nl (E.J.M.F.); inge.brouwer@wur.nl (I.D.B.); 2 Department of Family and Consumer Sciences, Faculty of Agriculture, University for Development Studies, P.O. Box TL 1882, Tamale, Ghana; 3 Department of Nutritional Sciences, School of Allied Health Sciences, University for Development Studies, P.O. Box TL 1883, Tamale, Ghana; abdul-razak.abizari@uds.edu.gh or; 4 Partnership for Child Development (PCD), Department of Infectious Disease Epidemiology, Imperial College London, London W2 1PG, UK; e.aurino@imperial.ac.uk; 5 International Food Policy Research Institute (IFPRI), 1201 Eye Street NW, Washington DC 20005-3915, USA; A.Gelli@cgiar.org; 6 Micronutrient Forum, 10th Floor, 1201 Eye St NW, Washington DC 20005, USA; 7 Department of Social Sciences, Sociology of Consumption and Households, Wageningen University & Research, 6708 PB Wageningen, The Netherlands; h.a.j.bras@rug.nl

**Keywords:** stunting, underweight, overweight, pre-hypertension/hypertension, adolescents, Ghana

## Abstract

The sex differences in malnutrition and hypertension during adolescence is largely inconclusive. There is also a paucity of data on the sex-specific correlates of malnutrition and hypertension for adolescents. Hence, this study aimed to assess the association between malnutrition, pre-hypertension/hypertension (PHH) and sex among adolescents. The study also aimed to determine and contrast the factors associated with these risks in Ghana. We analysed data of non-pregnant adolescent girls (*n* = 857) and adolescent boys (*n* = 870) aged 15–19 years from the 2014 Ghana Demographic and Health Survey (DHS). We modelled the prevalence risk ratio (PRR) of malnutrition and PHH using Cox proportional hazard models. Compared to adolescent girls, boys were more than twice likely to be stunted (PRR = 2.58, 95% C.I (1.77, 3.76)) and underweight (PRR = 2.67, 95% C.I (1.41, 5.09)) but less likely to be overweight/obese (PRR = 0.85, 95% C.I (0.08, 0.29)). Boys were also about twice likely to have PHH (PRR = 1.96, 95% C.I (1.47, 2.59)) compared to their female peers. Girls were more at risk of the detrimental effects of poor education on stunting and PHH. Empowerment index while protective of stunting for girls (PRR = 0.82, 95% C.I (0.67, 0.99)) also increased their risk of overweight/obesity (PRR = 1.31, 95% C.I (1.02, 1.68)). A higher household wealth index (HWI) increased the risk of overweight/obesity for adolescent girls but was protective of stunting and PHH for adolescent boys. Improvement in household water, hygiene, and sanitation (WASH) reduced the risk of stunting by 15% for adolescent boys. Overall, our findings suggest a double-burden of malnutrition with an up-coming non-communicable disease burden for adolescents in Ghana. Our findings may also be highlighting the need to target adolescent boys alongside girls in nutrition and health intervention programmes.

## 1. Introduction

There are more adolescents in the world than ever, particularly in South-East Asia and Sub-Saharan Africa and the life stage of adolescence (approximately from 10–19 years) is receiving growing attention in international development [1]. About a quarter of the Ghanaian population is adolescents (aged 10–19 years) with the proportion of male adolescents (23.2%) slightly higher than female adolescents (21.7%) [2]. Adolescence is a period of rapid growth and maturation from childhood to adulthood, second to the 1000 days of life [3]; this period is characterised by an increase in nutrient requirements [4].

The association between malnutrition and sex is inconclusive, mainly [5]. However, Abarca-Gómez et al. [6] in their analysis of the worldwide trend in body-mass index (BMI), showed that 5–19 years girls in many sub-Saharan African countries have consistently had a higher BMI than their male counterparts from 1975 to 2016. Furthermore, Manyanga et al. [7], in their analysis of the Global School Health Survey data for Africa, reported a higher prevalence of overweight for adolescent girls compared to boys in all countries except for Egypt and Malawi. On the contrary, adolescent boys had a higher prevalence of underweight than girls for every country in the analysis of Manyanga et al. [7].

In the 2014 Ghana Demographic and Health Survey (GDHS) report [8], 15–19 years adolescents boys compared to their female peers were reported to have a higher prevalence of stunting and underweight while the girls had a higher overweight prevalence. Emerging data from Ghana has also shown varied and relatively higher prevalence rates of stunting (15–50.3%), underweight (7–19.4%), and overweight/obesity (6.9–17%) among adolescent males and females depending on the context in which the study was conducted [9,10,11,12]. Although these studies are limited by their cross-sectional design and geographical scope, which mainly includes the metropolitan areas of Accra and Kumasi; the general conclusion is that Ghanaian adolescent boys are often worse off regarding stunting and underweight compared to their female peers. In contrast, adolescent girls are said to be more at risk of overweight in Ghana compared to their male colleagues.

Literature from Ghana [9,10,11] has identified poor parental education as a factor associated with undernutrition for adolescents. Moreover, poor sanitation, inadequate dietary intake and household food insecurity are well-known correlates of stunting and underweight among adolescents [5]. While rural residence is linked to undernutrition, residing in an urban setting has been linked with a higher likelihood of overnutrition [5,6,7]. Similarly, poor socioeconomic status is positively associated with undernutrition but inversely associated with overnutrition [5,6,10,11]. The increasing burden of overweight and obesity has also been attributed to the increasing consumption of “fast foods” loaded with fats, increasing consumption of sugar-sweetened carbonated drinks, and sedentary lifestyles [10,12,13,14]. In addition to physical activity [12,15] and dietary habits [14], which are known to be poorer in adolescent girls in Ghana, no difference between males and females in the other correlates of malnutrition above have been documented for adolescents.

Regardless of the sex differences in under and over-nutrition, while the persisting rates of undernutrition undermine the development of Ghanaian adolescents into adulthood, the evolving burden of over nutrition poses an emerging cardiovascular risk for them [16]. Though there is a scarcity of data on cardiovascular risk among adolescents in Ghana, two cross-sectional studies in the Greater Accra and Ashanti regions reported a higher prevalence of hypertension and pre-hypertension among adolescent males compared to females [10,12]. Family history of hypertension, low birth weight, and excess weight are known determinants of hypertension [17]. In a study by Afrifa-Anane et al. [12] in Ghana, low physical activity and a higher BMI were associated with higher systolic blood pressure.

To the best of our knowledge, no study has examined the sex-specific correlates of the nutritional status or the cardiovascular risk of female adolescents compared to their male colleagues in Ghana. The current evidence, as presented, is mostly based on a few cross-sectional studies with relatively smaller sample sizes [9,10,11,12]. Age and sex-specific data on the health and nutritional status of adolescents at the national level are urgently needed for policies and programmes for improving the health and nutritional status of adolescents [18]. Hence, the primary objective of this study was to assess the association between malnutrition, pre-hypertension/hypertension (PHH) and sex among adolescents. The study also aimed to determine and contrast the associated factors among the adolescents in Ghana using national representative data from the 2014 Ghana DHS. While contributing to the literature in the sex differences in malnutrition and cardiovascular risk for adolescents, our analyses may help explain the factors moderating the differences.

## 2. Materials and Methods

### 2.1. Study Design

The 2014 Ghana DHS data were analysed in this study. The Ghana DHS used a multistage random sampling approach, including both rural and urban areas in all the regions of Ghana. In the multistage sampling, clusters were first selected randomly, followed by a random selection of a community in each cluster; all households in the community selected were interviewed. The 2014 Ghana DHS data included anthropometric data (weight and height) as well as data on systolic and diastolic blood pressure of both adolescent females and males aged 15–19 years, as well as data on demographics, fertility, household characteristics, nutrition, and health. These data are in the public domain and available from the DHS MEASURE website, and details about the surveys can be found in the DHS Methodology report [19]. The Ghana Health Service Ethical Review Committee, Accra, Ghana approved the DHS, and the data was collected by the Ghana Statistical Service and the Ghana Health Service. We obtained permission from “DHS MEASURE’’ to download and analyse the data, and no further ethical approval was required because anonymous identifiers were used in coding the DHS data. The population for analyses included non-pregnant adolescent girls (*n* = 857) and adolescent boys (*n* = 870) aged 15–19 years.

### 2.2. Malnutrition and Hypertension

#### 2.2.1. Anthropometric Indicators

We computed height-for-age *z*-score (HAZ) and body-mass-index for-age *z*-score (BAZ) using WHO AnthroPlus (version 1.0.4). We defined stunting (HAZ < −2SD), underweight (BAZ < −2SD), normal weight (−2SD ≤ BAZ ≤ +1SD), overweight (+1SD < BAZ < +2SD), and obesity (BAZ ≥ +2SD) as recommended [20].

#### 2.2.2. Blood Pressure and Hypertension

Systolic blood pressure (SBP) and diastolic blood pressure (DBP) were measured in triplicates in the Ghana DHS. We computed and used the mean of SBP and as well as DBP in our analysis of blood pressure (BP) and cardiovascular risk. We defined cardiovascular risk as pre-hypertension or hypertension (PHH). In conformity with the US National Heart, Lung, and Blood Institute’s recommended cut-off points, we defined hypertension as SBP and/or DBP ≥ 95th percentile for sex, age and height [21]. Pre-hypertension was also defined as BP ≥ 120/80 mmHg and/or SBP/DBP ≥ 90th percentile but <95th percentile for sex, age, and height [21].

### 2.3. The Conceptual Framework for the Analyses

The theoretical framework for our analysis was based on Madjdian et al. [5] and Lassi et al. [22]. The framework of Madjdian et al. (2018) postulates that border community-level factors such as type of residence and ecological zone exert an influence on the household characteristics which tend to affect some of the individual-level determinants of nutrition and health. The framework of Lassi et al. (2017) asserts that women empowerment, water, and sanitation are mediating factors in improving the nutrition of adolescents and young women.

#### 2.3.1. Individual-Level Variables

Variables analysed at the individual level included age, religion, ethnicity, education assessed as the number of completed years of schooling as well as categorical, marital status and, the relationship of the adolescent to the household head. We also included dichotomous variables for whether the adolescent ever had a child, and experienced sex. The variables for the working life of the adolescent included a categorical variable for the type of occupation and a dichotomous variable for any paid work in the past year. Lifestyle factors in the analysis included the frequency of watching TV and of listening to the radio in the past week. Furthermore, the analysis included health-seeking behaviour factors, including having visited a health facility in the last 6 months, sleeping under a mosquito net, and being covered by the Ghana National Health Insurance Scheme (NHIS). Data on dietary intake included the frequency (days) of the consumption of fruits and of vegetables in the past week. Covariates for BP assessment in the data included binary variables (yes/no) for any form of physical activity, smoking, coffee intake, and food intake 10 min before the measurement. The data also included the household consumption of salty foods in the last 24-h including (1) broth cubes added to food, (2) processed canned meat/fish/legume, (3) salted dried fish/koobi/kako, and (4) any other salty ingredients. A summated score comprising the four listed salty food items denoted the household salty food consumption (SAFC); a proxy for the salt intake of our sample. In the SAFC, a score of 1 else 0 was assigned if the household consumed any of the listed salty food items in the last 24-h. The SAFC ranged from 0 to 4; a higher score reflecting a higher salty food consumption by the household.

We created indexes of attitude towards wife-beating as well as of property ownership of the adolescent to reflect autonomy and empowerment similar to Amugsi and colleagues [23]. In the attitude towards wife-beating index, a score of 1 else 0 was assigned to a "no" answer to the following questions; wife-beating or hitting is justified if she (1) “goes out without telling him”, (2) “neglects the children”, (3) “argues with him”, (4) “refuses to have sex with him”, and (5) “burns the food”. A higher score for the adolescent girl meant she was more empowered; but for adolescent boys, it implied, he was more caring and tolerable. Similarly, in the index of property ownership, a score of 1 else 0 was assigned if the adolescent was allowed to own (1) land or (2) a house alone or jointly. The attitude toward wife beating index ranged from 0 to 5 while the property ownership index ranged from 0 to 2. Lastly, a summated empowerment index combined the attitude towards wife-beating and property ownership indexes; the index ranged from 0 to 7 with a higher score implying more empowered. Supplementary Table S1 illustrates the empowerment index.

#### 2.3.2. Household-Level Variables

Household related covariates included the household size, number of children under-five years in the house, the number of persons in the household covered by the NHIS, the age and sex of the household head, and the socioeconomic status of the household defined by the wealth index quintile of the household. In brief, the household wealth index (HWI) in the DHS was computed with the household’s ownership of selected assets, such as TV and bicycles; materials used for housing construction; and types of water access and sanitation facilities and cooking fuel [19]. Furthermore, dichotomous variables for the household ownership of land and farm animals were included. In the analysis, we also computed and used the mean years of education of household members as a continuous variable.

Lastly, based on the available data, we constructed a composite index of improved household water, hygiene, and sanitation (WASH), which ranged from 0 to 8 by aggregating scores for an improved sanitation index (SI), improved water index (WI), and improved household hygiene index (HHI). The WASH followed the WHO/UNICEF (2006) [24] guidelines on improved household water and sanitation in the prevention of oral–faecal contamination. The SI ranged from 0 to 2, whereby access to an improved toilet facility was scored 1 else 0, household not sharing the toilet facility with others was scored 1 else 0. The WI also ranged from 0 to 3 with a score of 1 else 0 assigned if (1) the household has access to an improved source of drinking water, (2) the round-trip water haulage time between the household and water source was ≤30 min, and (3) water was treated before drinking by the household. Lastly, the HHI ranged from 0 to 3 and included a score of 1 else 0 for the availability of (1) a place for washing hands in the household, (2) water for washing hands in the household, and (3) soap and detergents for washing hands in the household. Similar indexes have been used in previous studies [25,26]. The details of the WASH index can be found in the online Supplementary Material (Table S3).

#### 2.3.3. Broader Community and Environmental Variables

The region of residence was recoded into 3 agro-ecological zones [27] for the analyses including (1) Guinea/Sudan savannah (Northern, Upper East, and Upper West Regions), (2) coastal savannah (Central, Greater Accra, and Volta Regions), and (3) Forest zone (Brong Ahafo, Ashanti, Western, and Eastern Regions). The type of residence (rural or urban) was also included in the analysis. The DHS data defined rural areas as countryside while town and cities were classified as urban [19]. Finally, we included a dichotomous variable for access to a health program for adolescents accessing health services in our analysis.

### 2.4. Statistical Analysis

Descriptive statistics were presented as percentages for dichotomous/categorical variables and as means (standard errors) for continuous variables. Differences between groups in continuous variables were tested using one-way ANOVA (GLM function in SAS) or the Mann–Whitney test where necessary (for skewed data). In contrast, differences between groups for dichotomous/categorical variables were tested with the Rao–Scott Chi-Square Test. Cox proportional hazard models (stunted vs. not stunted, underweight vs. normal weight, overweight/obese vs. normal weight, and PHH vs. normotensive) using “PROC SURVEYPHREG” in SAS were subsequently fitted to compare the risks and to identify the predictors of malnutrition and cardiovascular risk status. The effect measured was the prevalence risk ratios (PRR). Cox and Poisson models have more robust variance and are thus better alternatives than logistic regression in cross-sectional data analysis [28,29]; explaining our choice of PRR with Cox proportional hazard models.

In stratified bivariate analyses by sex, we determined the univariate associations of the different independent variables with the outcome variables. Variables from the bivariate analysis with *p*-values ≤ 0.25 were subsequently included in multivariable models for further assessment. Finally, we conducted a pooled analysis for both males and females. In the pooled analysis, interaction terms of sex and each of the first-order variables were included, and all interaction terms with *p*-values ≤ 0.1 were included in the multivariate models for further assessment. The final models were selected based on the log-likelihood ratio test, Akaike information criteria (AIC), Wald test, and *p*-value. The variance inflation factor (<10), as well as the Pearson and Spearman correlation coefficients, were used to assess multicollinearity in linear regression models. As a result of the complex survey design, we applied the weighting factors in the DHS data and adjusted for strata and cluster effects in all the analyses by using the “PROC SURVEY”, function in SAS [30]. A detailed explanation of the weighting procedure can also be found in the DHS Methodology report [19]. All statistical analyses were conducted with SAS 9.4 (SAS Institute Inc., Cary, NC, USA) and a two-tailed *p*-value ≤ 0.05 at 95% confidence interval was considered statistically significant. As a sensitivity analysis, we repeated all the analysis with linear regression using the “PROC SURVEYREG” command in SAS. In the sensitivity analyses, HAZ, BAZ, SBP, and DBP were the outcome variables; the results of the sensitivity analyses are presented as online Supplementary Material (Tables S4 and S5).

## 3. Results

### 3.1. Population Characteristics

The mean age of female adolescents was 16.8 (SE 0.05) years, while that of male adolescents was 17.0 (SE 0.05) years (Table 1). Furthermore, the proportion of adolescent girls who were unemployed was higher compared to adolescent boys (64.5% vs. 51.2%; *p* = 0.0001) (Table 1). Table 1 also shows that the frequency of listening to the radio (1.6 vs. 1.2, *p* < 0.0001), watching TV (1.4 vs. 1.2; *p* < 0.0001) were higher among males compared to females. However, more adolescent girls visited a health facility in the last six months before the survey compared to adolescent boys (19.2% vs. 10.8%; *p* < 0.0001). The results also showed that more adolescent girls were presently married compared to their male peers (5.7% vs. 0.5%; *p* <0.0001) (Table 1). The frequency of fruit consumption was higher for girls, but the frequency of vegetable consumption was somewhat higher for boys. The adolescent boys also had a higher frequency of watching TV and of listening to the radio. Overall, about a tenth of the adolescents engaged in some exercise ten minutes before the BP assessment with the proportion of boys marginally higher compared to the girls (15.4% vs. 9.7%; *p* = 0.01). Similarly, less than 1% drunk coffee ten minutes before the BP measurement, with only 0.2% of the boys having smoked.

Additionally, about 3.6% of the adolescent girls were spouses of the household heads. Moreover, more adolescent girls had experienced first sex compared to adolescent boys (41.1% vs. 26.7%; *p* < 0.0001) and more adolescent girls ever had a child compared to boys (10.8% vs. 0.7%; *p* < 0.0001). Of the adolescents who had experienced sex, the mean age for boys was lower compared to girls (14.8 vs. 15.3 years; *p* = 0.001) (Table 1). Nevertheless, the mean age for adolescents’ girls who ever had a child was lower compared to boys (16.59 vs. 18.15 yrs.; *p* = 0.025). With regards to autonomy and empowerment of the adolescent at the household, the mean autonomy and empowerment index score was higher for males compared to females (4.55 vs. 3.23; *p* < 0.0001). There were more adolescent males in male-headed households compared to their female colleagues (69.4% vs. 59.6%; *p* = 0.001). The average household size of the sample was about 5.7 (SE 0.10), and the mean age of household heads for the sample was 49.3 years (SE 0.55); none of these differed by the adolescent sex (Table 1). Table 1 also shows that the mean number of children under-five in households of adolescent girls was significantly higher than that of adolescent boys (*p* = 0.022). There were no differences in ethnicity, education, HWI, SAFC, mean household years of education, place of residence, agro–ecological zone, and the proportion of those covered by the NHIS between the adolescent boys and girls (Table 1).

### 3.2. Malnutrition and Cardiovascular Risk

**Figure 1:**
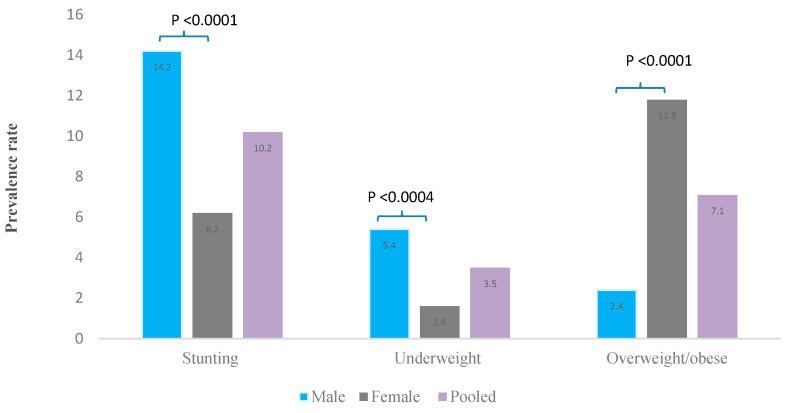
Comparison of the prevalence rates of chronic-energy malnutrition between 15–19 years adolescent boys and girls in Ghana.

Adolescent girls had a higher mean mid-upper arm circumference (MUAC) (26.3 vs. 25.6 cm; *p* < 0.0001), BAZ (0.03 vs. −0.59; *p* < 0.0001), and HAZ (−0.64 vs. −1.01; *p* < 0.0001) compared to their male colleagues (Table 1). Adolescent males were more stunted (14.2% vs. 6.2%; *p* < 0.0001) and underweight (5.4% vs. 1.6%; *p* = 0.0004) but less overweight/obese (2.4% vs. 11.8%; *p* < 0.0001) compared to their female peers (Figure 1).

**Figure 2:**
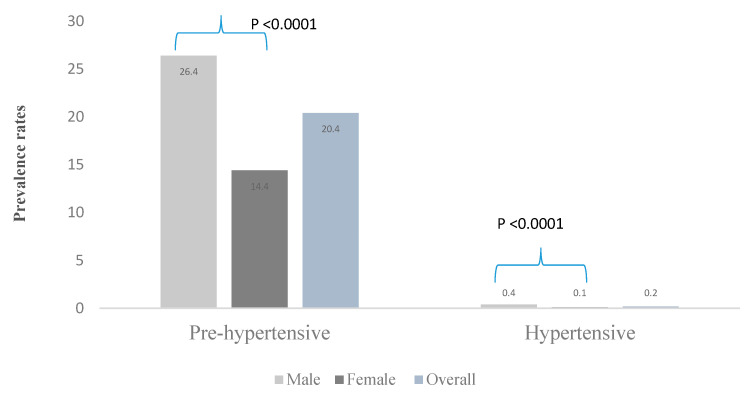
Comparison of the prevalence rates of pre-hypertension and hypertension between 15–19 years adolescent boys and girls in Ghana.

Moreover, adolescent males had a higher SBP (113.5 vs. 107.5 mmHg; *p* < 0.0001) but a lower DBP (67.8 vs. 69.2 mmHg; *p* < 0.0001) compared to their female peers (Table 1). Figure 2 shows that about a fifth of the adolescents were either pre-hypertensive or hypertensive. The proportion pre-hypertensive (26.4% vs. 14.4%) and hypertensive (0.4% vs. 0.1%) was higher for male compared to female adolescents.

The Cox regression models (Table 2) show that compared to adolescent females, adolescent males were more than twice likely to be stunted (PRR = 2.58, 95% C.I (1.77, 3.76)) and as well underweight (PRR = 2.67, 95% C.I (1.41, 5.09)). However, adolescent males were 85% less likely to be overweight/obese compared to their female peers (Table 3). Moreover, compared to adolescent females, adolescent males were about twice more likely to be PHH (PRR = 1.96, 95% C.I (1.47, 2.59)) (Table 3).

### 3.3. Factors Associated with Malnutrition

Compared to having secondary/higher education, no education and primary education were consistently associated with a higher risk of stunting for both adolescent boys and girls, but the association was stronger for girls than boys (Table 2). Similarly, compared to the fifth quintile of the HWI, adolescent boys in the first three quintiles were more likely to be stunted in all models. In the pooled model, the association between the HWI and stunting was attenuated with only the second quintile risk remaining statistically significant. Table 2 also shows that a unit increase in the WASH index was associated with a 15% lower risk of stunting for adolescent boys. Adolescent boys from the Akan ethnicity were meaningfully more likely to be stunted compared to those from other ethnicities (PRR = 1.64, 95% C.I (1.10, 2.44)). Among, adolescent girls, the risk of stunting was markedly lower for currently married girls compared to never-married girls (Table 2).

Furthermore, girls who menstruated in the last six weeks preceding the survey compared to those who did not were 77% less likely to be stunted (PRR = 0.23, 95% C.I (0.11, 0.48)). Moreover, the risk of stunting was significantly higher for adolescent girls for a unit increase in age. While a unit increase in the frequency of vegetable consumption in the past week was associated with an increased risk of stunting for girls, a unit increase in the frequency of fruit consumption in the last week seemed protective for them. In the pooled model, the effect of the frequency of vegetable consumption was still significant though attenuated. Lastly, a unit increase in the empowerment index was protective of stunting in the statistical model for the adolescent girls and remained significant in the pooled model, although attenuated (Table 2).

Table 2 also shows that fruit consumption (PRR = 0.75, (95% C.I (0.57, 0.98)] and menstruation six weeks before the survey (PRR = 0.18, (95% C.I (0.06, 0.54)) were protective of underweight adolescent girls. In the pooled model and that for adolescent boys, the risk of underweight was significantly lower when the adolescent had experienced sex compared to those who were yet to experience sex. Compared to being a daughter/son of the household head, the risk of underweight was significantly higher when the adolescent was a grandson (PRR = 2.12, 95% C.I (1.06, 4.24)) but significantly lower when the adolescent was a non-family relation in the pooled model (PRR = 0.11, (95% C.I (0.02, 0.86)] (Table 2). Furthermore, a unit increase in household size increased the risk of underweight by 8% for adolescent boys. Lastly, adolescent boys with primary school education compared to those with secondary/higher education were about twice likely to be underweight; in the pooled model, the association was attenuated.

Overall, the risk of overweight/obesity was consistently lower for adolescents from households in the first three quintiles of the HWI compared to the fifth quintile in all statistical models, and the association was strongest for girls but not boys (Table 3). Table 3 further shows that married adolescent girls had a higher risk of overweight/obesity compared to never-married adolescent girls (PRR = 3.53, 95% C.I (1.86, 6.69)). The risk of overweight/obesity increased by about 30% for a unit increase in the empowerment index in the pooled model, and in the model for adolescent girls (Table 3). Compared to adolescent boys in the forest zone, those in the coastal savannah zone seemed to have a lower risk of overweight/obese (PRR = 0.34, 95% C.I (0.11, 1.00)).

### 3.4. Factors Associated with Cardiovascular Risk

Table 3 also shows that the risk of PHH was significantly lower among adolescent girls for a unit increase in the frequency of watching TV in the past week and when the girl ever had a child compared to those who never had. However, adolescent girls who had primary education compared to those who had secondary/higher education were more likely to be PHH (PRR = 1.59, 95% C.I (1.01, 2.48)). An interaction term of sex and education in the pooled model showed that the effect of education was only significant for adolescent girls compared to boys. Ownership of agriculture land by the household appeared protective of PHH for adolescent girls. Furthermore, overweight adolescents were consistently more likely to be PHH compared to normal-weight adolescents in all statistical models (Table 3). In the pooled model as well as the model for adolescent boys, the risk of PHH was significantly higher for those in the poorest HWI compared to the wealthiest HWI. Similarly, adolescent boys in the Guinea/Sudan Savannah zone compared to the forest zone were significantly less likely to be PHH; the association remained in the pooled model, although attenuated. Finally, the consumption of coffee 10 min beforehand, the survey increased the risk of PHH by more than three times for adolescent boys. 

### 3.5. Model Fit and Sensitivity Analysis

The model fit statistics showed that all models were statistically significant. Lastly, the sensitivity analysis using linear regression showed a similar trend in the risk among adolescent boys compared to girls as well as the factors associated with malnutrition and PHH among adolescents (Tables S4 and S5). Notably, the interaction terms for HWI and sex was statistically significant for HAZ and SBP with boys having significantly lower HAZ and a higher SBP for the lower HWI quintiles compared to the highest quintile. Likewise, girls who ever had a child had a significantly lower HAZ, and a unit increase in the empowerment index increased the HAZ of girls significantly. Lastly, while the experience of sex increased the SBP of girls significantly, the consumption of coffee by boys also increased their SBP significantly.

## 4. Discussion

The present study compared the risk of malnutrition and PHH among adolescent boys and girls and the factors associated with these risks in Ghana. Overall, adolescent boys were more than twice likely to be stunted and underweight compared to their female peers. Madjdian and cohort [5] found that studies with a significant association between nutritional status and sex point to adolescent boys being worse off than girls in terms of stunting and underweight; partly attributed to boys’ later and "*sudden*" growth spurt [31]. In addition, males are also known to have a high risk for infections [32]; a higher infections risk may increase their risk of undernutrition and may partly account for our findings. Intra-household food distribution in Ghana does not favour girls [33] but Dapi et al. [34] argue that girls are often involved in cooking and shopping and might eat in between meals and during cooking; resulting in better nutritional intake for girls compared to boys.

In conformity with several other studies [7,9,11,34], adolescent males were less likely to be overweight/obese compared to their female colleagues. Generally, adolescent girls in Ghana are known to have less physical activity [12,15] and to have unhealthier eating habits than boys [14]; partially explaining our finding. According to Moubarac et al. [13], the increasing consumption of nutrient-poor, energy-dense snacks, highly processed foods, sugar-sweetened beverages, and convenient roadside foods (fast-foods) are linked with the emergence of overweight and obesity; this may also partially account for our finding. In the present study, we could not substantiate the findings of Moubarac et al. [13] as the 2014 DHS data did not include food intake or snacking habits. Nevertheless, a recent study in the Bekwai Municipality of Ghana indicated that snacking before bed increased the odds of overweight/obesity by more than ten times for 5–17 years children; girls were more overweight (19.4% vs. 7.6%, *p* < 0.001) and obese (10.2% vs. 7.3%, *p* = 0.177) compared to boys in this study [35]. The data on physical activity in the present study may not well reflect the usual physical activity behaviour of our sample. Accordingly, it was not surprising that physical activity did not appear as an important factor in any of our models. Even so, more adolescents boys were found to engage in some form of physical activity ten minutes prior to the BP assessment. In general, the cultural context in Ghana does not promote much aerobic outdoor games for girls.

Overall, Ghana is presently in the nutrition transition, mirrored in the changing dietary behaviours with more consumption of refined foods, especially in the urban areas [36]. This may partly account for the emergence of overnutrition in our sample. Although a higher frequency of the consumption of fruits and vegetables in the last week may partly explain healthy dietary behaviour, none were essential correlates of overweight/obesity in our models. However, a unit increase in the frequency of fruit consumption was protective of underweight and stunting for girls. Remarkably, an inverse association was found for the frequency of vegetable consumption and stunting for girls. In Ghana, fruits are often consumed as snacks and desserts, but vegetables are commonly consumed as soups and sauces alongside the staple foods. Hence, a high frequency of vegetable intake may represent compensation for reduced calorie intake, especially in settings where food insecurity is prevalent. For instance, in a group of eighth-grade children in the USA, Howard [37] found that children with very low household food security consumed a significantly higher number of servings of carrots. Similarly, Stevens and colleagues [38] reported that the peak of household food insecurity corresponded with higher consumption of dark green leafy vegetables and vitamin A-rich fruits/vegetables in a group of Bangladeshi pregnant women. The former may also explain that boys had a higher frequency of vegetable consumption as their calorie requirements are higher. Nonetheless, fruits and vegetables are rich sources of vitamins and minerals and are linked to improved health outcomes [39].

Considering that over a tenth of the adolescent girls in our study were either overweight or obese, there is a need to institute intervention programmes for the prevention of overnutrition for adolescents, particularly girls. Adolescent overweight/obesity is a known risk factor of obesity in adulthood and diabetes, hypertension, asthma, lower extremity venous oedema, and obstructive sleep apnoea in later life [40]. In our study, being overweight/obese increased the risk of PHH for both adolescent boys and girls. The mechanism of hypertension in overweight/obesity seems to be related to increased sodium sensitivity and the increment of sympathetic nervous system activity [17]. The only data in the DHS on salt intake for our sample related to the household consumption of salty foods in the last 24-h, which was not an important covariate of BP or PHH in our analysis. The SAFC in our analysis was only a proxy of the salt intake of our sample and did not sufficiently represent individual salt intake. Although the SAFC included some commonly consumed salty foods in the Ghanaian context, it fails to include all salty foods extensively, quantify the amount of salt consumed from these foods and as well capture the habitual intake of these salty foods. Of particular interest are modifiable factors such as high alcohol intake and high levels of substance use [17], which we could not verify in our data. Considering that only a small fraction (0.2%) of the adolescent boys smoked ten minutes prior the BP assessment, smoking behaviour may well not be an important covariate for our sample; it was thus neither a correlate of BP nor PHH in our analysis. Even physical activity ten minutes before the BP assessment was not a relevant correlate of BP in our present study, which may yet be that it did not reflect their usual physical activity level. Overall, the positive association between overweight/obesity and PHH in our analysis emphasises the need for public health intervention programmes to curtail overnutrition.

Interestingly, despite the 85% lower risk of overweight/obesity for adolescent boys, they were found to have a higher average SBP, with a higher risk of PHH compared to their female peers; confirming the findings Afrifa–Anane et al. [12] in Ghana. According to Abeelen et al. [41], inadequate maternal dietary intake during pregnancy is associated with higher hypertension risk among men but not women; they maintain that the nutrition of boys in utero is more dependent on the mother’s diet in pregnancy. Lassi and colleagues in Bundy et al. [42] also argue that stunted children and adolescents have higher rates of later arterial hypertension. They argue that undernutrition in childhood and adolescence results in constant physiologic and psychologic stress, increasing the production of stress hormones that weaken the body and decreasing the production of thyroid hormones and insulin-like growth factor that regulates growth. In a follow-up study in the Netherlands, Rotteveel and colleagues [43] showed that young adults born preterm have lower insulin sensitivity and higher blood pressure; low-birthweight and being born preterm are both determinants of stunting and underweight [44]. The above may partly account for the increased risk of PHH for adolescent males in our present study since they also had a higher risk of stunting and underweight.

Albeit, the proportion who consumed coffee ten minutes prior the BP assessment was slightly higher for girls compared to boys (1.1% vs. 0.4%, *p* = 0.16), adolescent boys who consumed coffee ten minutes before the assessment had a higher BP and an increased risk of PHH. Caffeine increases systemic vascular resistance through its ability to block adenosine receptors, and thereby increasing BP [45]. Furthermore, the total amount of caffeine rather than whether the adolescent consumed coffee or caffeine is more relevant [45]; however, we could not evaluate this with our data. TV watching is a known risk factor for overweight but was protective of PHH for adolescent girls in the present study. In our analysis, the frequency of TV watching was somewhat lower for girls compared to the boys and may account for the finding.

Conversely, it may be that girls who watched TV frequently were better informed about healthy behaviours. Gyamfi et al. [35] recently found TV watching protective of overweight/obesity in southern Ghana. Like Zimmerman and Bell [46], Gyamfi and colleagues [35] argued that the content of TV shows rather than TV watching itself is associated with poor nutrition outcomes since adolescents who watch educative programmes may be more educated. Nonetheless, we could not explore this further with the available data.

Our findings also suggest that girls are more at risk of the detrimental effects of poor education on stunting and PHH compared to their male peers. Generally, education is a proxy of socioeconomic status and empowerment [47], and more educated girls are more likely to be empowered. Adolescent girls who are more empowered can better take independent decisions, participate in key decision-making processes and have more control over household resources; significantly impacting her nutrition and health-seeking behaviours [23]. The preceding also explains that more empowered girls had a reduced risk of stunting in our study. Contrarywise, highly empowered girls, may have more purchasing power and may be more likely to consume the energy-dense, sugar-sweetened beverages and “fast-foods” earlier mentioned. The former comparatively accounts for the increased risk of overweight/obesity for a unit increase in the empowerment index for the adolescent girls in our analysis. In our analysis, a unit increase in the mean household years of education was associated with an increase in SBP for boys comparable to the HWI in our pooled model (results not shown). However, we excluded the mean household years of education from the SBP pooled model as it was correlated with the education of the adolescent and the HWI in particular; affirming that education is reflective of socioeconomic status. Additionally, girls who lived in households with agricultural land were less likely to be PHH, which may also be that such girls have easy access to agricultural land and are accordingly more economically empowered and less stressed in their livelihoods.

In the present study, a poor HWI was associated with an increased risk of stunting and PHH for boys but a reduced risk of overweight/obesity for girls in conformity with several other studies [5,6,7,10,11]. In general adolescents from poorer households are more likely to have inadequate dietary intake and with a low-purchasing power, may also be less likely to consume “fast-foods” and sugar-sweetened drinks and snacks. While persistent inadequate dietary intake may increase their risk of stunting, reduced calorie intake from the “fast-foods” and snacks may also reduce the risk of overweight/obesity. Contrariwise, those from low socioeconomic households may have poor access to health services, and may thus be more susceptible to poor health, including hypertension. The increased risk of stunting and PHH with a poor HWI for adolescent boys may as well be attributed to their susceptibility to poor health in such conditions. Improvements in household sanitation and hygiene are known to be associated with improved health and nutrition outcomes [48,49]. It was therefore not surprising that a higher WASH index was protective of stunting for the boys. While this finding may be affirming the susceptibility of males for infections [32], it also suggests improving WASH conditions may offer long-term beneficial effects for nutrition outcomes.

In our study, the risk of overweight/obesity was higher when a girl was formerly married compared to being never married. Considering the cross-sectional design or our analysis, the finding may be a reverse causality as studies have shown that heavier girls are more likely to be married earlier compared to their lighter peers [5]. Heavier girls may have better development of secondary sex characteristics, signalling readiness for sexual activity and childbearing [5,50].

Furthermore, the risk of overweight/obesity was lower for adolescent boys in the coastal savannah zone compared to the forest zone; which may slightly be explained by the differences in cultural dietary patterns, poverty, and food security by ecological zones in Ghana [51,52]. For instance, the Coastal Savannah zone is next to the Guinea/Sudan savannah zone in the prevalence of food insecurity [51] and poverty [52] in Ghana. In their analysis of food consumption patterns in Ghana, Ecker and Fang [36] observed that Ghana’s regions are at different stages of the nutrition transition and this was partly attributed to inequality in household income and food insecurity in the different regions. Lastly, adolescent males who were household heads were more likely to be PHH compared to those who were sons of the household head; this could somewhat be explained by the stress involved in catering for a household affecting their health negatively.

In addition to our present findings, adolescent girls remain a vulnerable group in Ghana with disadvantages in intra-household food distribution and resource allocation [33], sexual violence and exploitation [53], poor education [54], more substantial burdens in their time use and less income [55]. These deprivations are known to have intergenerational effects with the nutrition and health of the adolescent girl [56], which may explain that the focus of most nutrition and public health intervention programmes for adolescents in Ghana has mostly been on girls. Nevertheless, our findings may be emphasising the need to include adolescent boys alongside girls in nutrition and public health intervention programmes, ensuring a holistic improvement in the nutrition situation and consequently reducing the health care expenditure. Moreover, there is emerging evidence that there may be some paternal intergenerational effects on the health and nutritional status of the offspring [57].

Overall, the prevalence rates of malnutrition for this sample suggest a double burden of malnutrition with an upcoming diet-related non-communicable disease burden in Ghana. According to Hawkes et al. [58], a focus on the undernutrition burden of malnutrition may raise risks of poor-quality diets, obesity, and diet-related non-communicable diseases in countries undergoing a rapid nutrition transition. They argue that a more holistic approach is to simultaneously tackle both undernutrition and problems of overweight, obesity, and diet-related non-communicable [58]. Considering that Ghana is presently in the nutrition transition, this holistic approach including both under- and over-nutrition programs is required to make a meaningful impact; schools may be a viable place for such comprehensive school-based health intervention programmes. Wei et al. [59] recently reported the approach effective in reducing stunting and illness among 4–16 years Zambian children.

It was not feasible to map the trend, and determinants of malnutrition and cardiovascular risk among the adolescents in this analysis since only the 2014 survey included anthropometry of male adolescents aged 15–19 years. More so, data on BP was only available in the 2014 survey. Similarly, the present study did not compare anaemia risk as none of the DHS surveys has data on the haemoglobin status of adolescent boys. Additionally, the 2014 DHS did not include any data on dietary diversity; hence, we could not model dietary diversity in any of our statistical analyses. Nonetheless, the inclusion of the frequency of the consumption of fruits and vegetables in the past week may partly account for the adolescents’ dietary intake and dietary quality.

Some aspects of the DHS data, such as the frequency of the consumption of vegetables and fruits, may be subject to recall bias as this information was based on recalls. Although we could not account for this bias, the bias seems random and may be less likely to influence our conclusions. Inference of a possible causality in this analysis may be speculative since the DHS survey employed a cross-sectional study design. Hence, our findings are limited to the description of observed associations. Nevertheless, we thoroughly modelled several potential explanatory variables, including subject and household-related variables through robust statistical models with weighting, while accounting for the complex survey structure in the DHS data using the PROC SURVEY function in SAS [30]. Lastly, the use of two different statistical models as in Cox proportional hazard models and linear regression ensured that we minimised any methodological-related biases while accounting for all possible explanatory variables that were available.

## 5. Conclusions

The present study has shown that Ghanaian adolescent boys are more than twice likely to be stunted, underweight, and pre-hypertensive/hypertensive compared to their female peers. However, adolescent girls were more likely to be overweight/obese compared to their male colleagues. Girls were more at risk of the detrimental effects of poor education on stunting and PHH. Empowerment, as measured by the empowerment index while protective of stunting for girls, also increased their risk of overweight/obesity. Our findings indicate that improving HWI increases the risk of overweight/obesity for adolescent girls but reduces the risk of stunting and PHH for adolescent boys. Improvement in household WASH conditions may have more beneficial effects on the nutrition of adolescent boys.

Overall, the prevalence rates of malnutrition and PHH for this sample suggest a double burden of malnutrition with an up-coming non-communicable disease burden and a holistic approach including both under- and over-nutrition programmes are required to make a meaningful impact. Notably, the positive association between overweight and PHH emphasises the need for public health intervention programmes to curtail overnutrition; such programmes may include the promotion of physical activity and healthy dietary behaviours. Our findings may also be highlighting the need to target adolescent boys alongside girls in nutrition and health intervention programmes.

## Figures and Tables

**Table 1:** Population Descriptive Statistics for adolescent boys and girls aged 15–19 years from the 2014 Ghana Demographic Health Survey Data.

Variables	Pooled Data (*n* = 1727)		Girls (*n* = 857)		Boys (*n* = 870)		*p*-Value ^1^
	Mean or Percentage	S.E (Mean)	Mean or Percentage	S.E (Mean)	Mean or Percentage	S.E (Mean)	
Nutritional status and blood pressure							
Arm circumference (cm)	26.00	0.12	26.31	0.18	25.68	0.17	<0.0001
Weight (kg)	54.17	0.25	53.42	0.32	54.91	0.39	<0.0001
Height (cm)	162.22	0.23	158.28	0.23	166.15	0.31	<0.0001
Age (years)	16.89	0.04	16.83	0.05	17.00	0.05	0.048
BMI (kg/m^2^)	20.54	0.07	21.30	0.11	19.78	0.09	<0.0001
BAZ	−0.28	0.02	0.03	0.03	−0.59	0.04	<0.0001
HAZ	−0.83	0.03	−0.64	0.03	−1.01	0.04	<0.0001
Systolic blood pressure (mmHg)	110.49	0.33	107.52	0.45	113.48	0.46	<0.0001
Diastolic blood pressure (mmHg)	68.52	0.25	69.22	0.39	67.83	0.31	<0.0001
Dietary intake and lifestyle							
Frequency of listening to radio in the past week	1.40	0.03	1.25	0.03	1.55	0.04	<0.001
Frequency of watching television in week the past week	1.32	0.03	1.21	0.04	1.42	0.05	<0.001
Frequency of fruit intake in the past week	2.97	0.09	3.04	0.11	2.90	0.12	0.001
Frequency of vegetable intake in the past week	3.58	0.10	3.26	0.11	3.89	0.14	<0.001
Health seeking behaviour							
Visited a health facility in the last 6 months ^2^	15.0		19.2		10.8		<0.0001
Slept under mosquito bed net ^2^			28.5		-		-
Covered by National Health Insurance Scheme (NHIS) (yes) ^2^	55.6		56.9		54.4		0.43
Blood pressure related covariates							
Eaten in the past 10 minutes ^2^	52.9		58.9		46.9		0.0001
Had coffee in the past 10 minutes ^2^	0.7		1.1		0.4		0.16
Smoked in the past 10 minutes ^2^	0.1		0		0.2		-
Exercised in the past 10 minutes ^2^	12.6		9.7		15.4		0.01
SAFC	1.55	0.03	1.58	0.04	1.51	0.05	0.70
Demographics							
Religion ^2^							0.003
Christian	78.7		80.6		76.8		
Muslim	16.9		17.1		16.6		
Other	4.4		2.3		6.6		
Ethnicity ^2^							0.21
Akan	50.6		49.3		51.9		
Mole-Dagbani	14.9		16.6		13.3		
Other	34.5		34.1		34.8		
Highest year of education	2.98	0.03	3.01	0.08	2.95	0.08	0.012
The highest educational level of adolescent ^2^							0.11
No education	3.2		3.8		2.5		
Primary school	22.1		23.7		20.5		
Secondary education/Higher	74.7		72.5		77.0		
Occupation of adolescent ^2^							0.0001
Unemployed	57.8		64.5		51.2		
Agriculture/unskilled labour	31.4		26.9		35.9		
Skilled labour	10.7		8.6		12.9		
The adolescent is currently in paid work ^2^	38.9		33.1		44.7		0.001
Decision making and empowerment indices							
Summated empowerment index	3.91	0.05	3.23	0.06	4.55	0.05	
Fertility, Marriage and Family Relations							
The adolescent has experienced sex already ^2^	33.9		41.1		26.7		<0.0001
Age at first sex	15.10	0.11	15.28	0.11	14.83	0.22	<0.001
The adolescent has a child ^2^	5.8		10.8		0.7		<0.0001
Age with first child	16.68	0.16	16.59	0.15	18.15	0.25	0.025
Total children adolescent has	1.14	0.05	1.16	0.06	1.00	0.00	<0.001
Relation of adolescent to household head ^2^							0.99
Household head	3.2		1.9		4.5		
Spouse	1.8		3.6		N/A		
Child (son/daughter)	66.3		62.4		70.2		
Grandchild	8.0		8.0		8.0		
Other family relation	14.1		15.5		12.6		
Non-family relation	6.6		8.6		4.6		
Marital status ^2^							<0.0001
Never married	96.6		93.6		99.5		
Currently married	3.1		5.7		0.5		
Formerly married	0.3		0.7		0		
Household characteristics of the adolescent							
Age of household head	49.34	0.55	49.20	0.67	49.48	0.83	0.59
Household size	5.74	0.10	5.70	0.11	5.78	0.13	0.57
Number of children < 5yrs	0.67	0.03	0.72	0.04	0.62	0.04	0.022
WASH index	3.57	0.07	3.57	0.08	3.56	0.09	0.96
Number of household members covered by health insurance	3.61	0.14	3.57	0.13	3.65	0.22	0.33
Sex of household head (male) ^2^	64.5		59.6		69.4		0.001
Household wealth index ^2^							0.27
Poorest	22.8		22.7		22.9		
Poorer	20.9		22.0		19.8		
Middle	19.2		18.8		19.6		
Richer	19.7		17.4		22.0		
Richest	17.4		19.1		15.7		
Household owns land usable for agriculture ^2^	49.1		46.1		52.2		0.04
Household owns livestock ^2^	50.5		48.6		52.4		0.26
Mean household years of education	5.42	0.12	5.28	0.16	5.56	0.13	0.22
Geographical/environmental							
Access to a health program for adolescents accessing health services ^2^	32.1		30.1		34.2		0.25
Place of residence ^2^							0.54
Rural	50.4		50.5		51.4		
Urban	49.6		49.5		48.6		
Agro–ecological zone ^2^							0.25
Forest	49.5		50.7		48.3		
Guinea/Sudan savannah	16.4		17.4		15.3		
Coastal savannah	34.1		31.9		36.4		

^1^ Between-group test for boys and girls; ^2^ Values are percentages; all other values are means and standard errors; BMI, Body mass index, BAZ, Body mass index for-age *z*-score; HAZ, height-for-age *z*-score; SAFC, household salty food consumption score; WASH, Water, Sanitation and Hygiene; N/A, not applicable as none of the adolescent boys was a spouse to the household head.

**Table 2:** Multivariate Predictors of Stunting and Underweight: A Comparative Analysis for Adolescent boys and girls from the 2014 Ghana Demographic Health Survey (GDHS) Data.

Variables	Stunting						Underweight					
	Girls (*n* = 857)		Boys (*n* = 870)		Pooled (*n* = 1727)		Girls (*n* = 857)		Boys (*n* = 870)		Pooled (*n* = 1727)	
	PPR	95% C.I	PPR	95% C.I	PPR	95% C.I	PPR	95% C.I	PPR	95% C.I	PPR	95% C.I
Sex												
Boy					2.58	1.77, 3.76 ****					2.67	1.41, 5.09 ***
Girl					Ref.						Ref.	
Age	1.42	1.19, 1.71 ****										
Girl Menstruated in the last 6 weeks												
Yes	0.23	0.11, 0.48 ****					0.18	0.06, 0.54 ***				
No	Ref.						Ref.					
The adolescent has experienced first sex												
Yes									0.38	0.15, 0.93 ***	0.49	0.23, 1.04 *
No									Ref.		Ref.	
Frequency of fruit intake in the past week	0.88	0.76, 1.01 *					0.75	0.57, 0.98 **				
Frequency of vegetable intake in the past week	1.13	1.01, 1.28 **			1.09	1.01, 1.17 **						
Empowerment index score	0.82	0.67, 0.99 **			0.90	0.81, 1.01 *						
Ethnicity												
Akan			1.64	1.10, 2.44 **								
Mole-Dagbani			0.97	0.62, 1.53								
Other			Ref.									
Relation of girl to the household head												
Household head									0.91	0.11, 7.17	0.48	0.07, 3.52
Granddaughter/grandson									2.12	1.06, 4.24 **	1.58	0.78, 3.21
Other family relation									0.63	0.23, 1.74	0.72	0.33, 1.57
Non-family relation									0.19	0.02, 1.46	0.11	0.02, 0.86 **
Daughter/son									Ref.		Ref.	
Marital status												
Currently married	0.24	0.08, 0.79 **										
Formerly married	N/A											
Never married	Ref.											
The highest educational level of girl												
No education	4.03	1.89, 8.60 ****	0.65	0.09, 4.57	1.80	0.87, 3.70			0.41	0.05, 3.17	1.14	0.33, 3.90
Primary school	1.81	0.90, 3.64 *	1.78	1.22, 2.59 ***	1.72	1.21, 2.45 ***			2.01	1.10, 3.66 **	1.82	1.08, 3.10 **
Secondary education/Higher	Ref.		Ref.		Ref.				Ref.		Ref.	
Household size									1.08	1.01, 1.17 **		
Water Hygiene and Sanitation index			0.85	0.73, 0.99 **								
Household wealth index												
Poorest			3.36	1.15, 9.81 **	1.90	0.86, 4.22						
Poorer			5.28	1.93, 14.43 ****	3.16	1.39, 7.19 ***						
Middle			4.17	1.48, 11.77 ***	2.21	0.98, 4.95 *						
Richer			2.42	0.85, 6.91 *	1.45	0.62, 3.38						
Richest			Ref.		Ref.							
**Model fit statistics**												
Wald test		28.45 ****		5.27 ****		7.37 ****		8.70 ****		3.90 ***		212.08 ****
-2 Log-likelihood ratio		213.09		510.81		824.31		54.10		192.83		277.25
AIC		231.09		528.81		842.31		58.10		208.83		295.25

PPR, prevalence risk ratio; 95% C.I, 95% confidence interval; Ref, Reference group; * *p* ≤ 0.1; ** *p* ≤ 0.05; *** *p* ≤ 0.01; **** *p* ≤ 0.001; N/A unreliable estimate as none of the formerly married was stunted. AIC, Akaike information criteria.

**Table 3:** Multivariate Predictors of Overweight and Pre/Hypertension: A Comparative Analysis for Adolescent boys and girls from the 2014 Ghana Demographic Health Survey (GDHS) Data.

Variables	Overweight/Obesity						Pre-Hypertension/Hypertension					
	Girls (*n* = 857)		Boys (*n* = 870)		Pooled (*n* = 1727)		Girls (*n* = 857)		Boys (*n* = 870)		Pooled (*n* = 1727)	
	PPR	95% C.I	PPR	95% C.I	PPR	95% C.I	PPR	95% C.I	PPR	95% C.I	PPR	95% C.I
Sex												
Boy					0.15	0.08, 0.29 ****					1.95	1.47, 2.59 ****
Girl					Ref.						Ref.	
Age									1.15	1.05, 1.26 ***	1.11	1.03, 1.19 ***
The adolescent has a child												
Yes							0.26	0.11, 0.61 ***			2.10	0.79, 5.61
No							Ref.				Ref.	
An interaction term for having a child * female sex											0.10	0.03, 0.38 ****
Frequency of watching television in the past week							0.72	0.57, 0.90 ***				
Empowerment index	1.31	1.02, 1.68 **			1.30	1.04, 1.62 **						
Had coffee in the past 10 min												
Yes									3.64	2.56, 5.19 ****		
No									Ref.		Ref.	
Relation of girl to the household head												
Household head									1.69	1.12, 2.54 ***		
Grand daughter									0.89	0.50, 1.61		
Other family relation									1.32	1.01, 1.74		
Non-family relation									0.74	0.31, 1.77		
Daughter/Son									Ref.			
Marital status												
Currently married	0.65	0.23, 1.86										
Formerly married	3.53	1.86, 6.69 ****										
Never married	Ref.											
The highest educational level of girl												
No education							0.50	0.15, 1.66			0.80	0.38, 1.67
Primary school							1.59	1.01, 2.48 **			1.04	0.76, 1.43
Secondary education/Higher							Ref.				Ref.	
An interaction term for highest educational level * sex												
Girl * No education											0.76	0.22, 2.12
Boy * Primary school											1.62	1.01, 2.62 **
Household owns land for agriculture												
Yes							0.59	0.39, 0.90 ***				
No							Ref.					
Sex of household head												
Male									0.75	0.57, 0.99 **		
Female									Ref.			
Household wealth index												
Poorest	0.21	0.10, 0.41 ****	0.21	0.03, 1.56	0.19	0.10, 0.36 ****			2.24	1.25, 4.03 ***	2.20	1.26, 3.83 ***
Poorer	0.15	0.07, 0.35 ****	0.07	0.01, 0.57 **	0.13	0.06, 0.29 ****			1.32	0.69, 2.53	1.48	0.79, 2.76
Middle	0.51	0.28, 0.93 **	0.15	0.03, 0.88 **	0.43	0.25, 0.74 ***			1.10	0.59, 2.06	1.25	0.76, 2.06
Richer	0.78	0.48, 1.28	0.59	0.14, 2.44	0.76	0.46, 1.25			1.60	0.87, 2.93	1.46	0.92, 2.32
Richest	Ref.		Ref.		Ref.				Ref.		Ref.	
Agro–ecological zone												
Coastal savannah			0.34	0.11, 1.00 **					1.00	0.75, 1.33	1.01	0.76, 1.32
Guinea/Sudan savannah			0.31	0.05, 2.15					0.55	0.37, 0.81 ***	0.61	0.42, 0.88 ***
Forest			Ref.						Ref.		Ref.	
BAZ category												
Underweight							0.86	0.21, 3.53	1.11	0.64, 1.92	1.00	0.61, 1.65
Overweight							1.69	1.06, 2.70 **	1.72	0.96, 3.07 *	1.76	1.20, 2.58 ***
Normal weight							Ref.				Ref.	
**Model fit statistics**												
Wald test		9.37 ****		3.20 ***		16.61 ****		4.65 ****		8.03 ***		5.00 **
−2 Log-likelihood ratio		417.67		77.80		540.65		528.98		984.81		1696.00
AIC		431.67		89.80		552.65		542.98		1014.81		1730.00

PPR, prevalence risk ratio; 95% C.I, 95% confidence interval; Ref, Reference group; * *p* ≤ 0.1; ** *p* ≤ 0.05; *** *p* ≤ 0.01; **** *p* ≤ 0.001; BAZ, body-mass-index for-age *z*-score.

## Supplementary Materials

The following are available online at https://www.mdpi.com/2072-6643/12/9/2737/s1, Table S1: Decision making score for adolescent males and females in DHS 2014 data, Table S2: Scoring format from responses for decision-making indexes; Table S3: Improved household water, hygiene and sanitation index (WASH), Table S4: Multivariate Predictors of Height-For-Age *Z*-Score (HAZ) and Body-Mass-Index-For-Age *Z*-Score (BAZ): A Comparative Analysis for Adolescent Boys and Girls from the 2014 Ghana Demographic Health Survey (GDHS) Data, Table S5: Multivariate Predictors of Blood Pressure: A Comparative Analysis for Adolescent Boys and Girls from the 2014 Ghana Demographic Health Survey (GDHS) Data.

## Abbreviations

BAZ	Body-Mass-index-for-Age *z*-score
BMI	Body Mass Index
BP	Blood Pressure
DBP	Diastolic blood pressure
DHS	Demographic and Health Survey
HAZ	Height-for-age z-score
HHI	household hygiene index
HSI	Household sanitation index
HWI	Household wealth index
PHH	Pre-hypertension/hypertension
SAFC	Household salty food consumption score
SBP	Systolic blood pressure
WASH	Water, hygiene and sanitation index
WI	water index

## References

[1] (2014). WHO Health for the World’s Adolescents: A Second Chance in the Second Decade.

[2] Ghana Statistical Service (GSS) (2013). 2010 Population and Housing Census Report. Children, Adolescents & Young People in Ghana.

[3] Black R.E., Victora C.G., Walker S.P., Bhutta Z.A., Christian P., De Onis M., Ezzati M., Grantham-Mcgregor S., Katz J., Martorell R. (2013). Maternal and child undernutrition and overweight in low-income and middle-income countries. Lancet.

[4] Mesías M., Seiquer I., Navarro M.P. (2013). Iron nutrition in adolescence. Crit. Rev. Food Sci. Nutr..

[5] Madjdian D.S., Azupogo F., Osendarp S., Bras H., Brouwer I. (2018). Socio-cultural and economic determinants and consequences of adolescent undernutrition and micronutrient deficiencies in LLMICs: A systematic narrative review. Ann. N. Y. Acad. Sci..

[6] Abarca-Gómez L., Abdeen Z.A., Hamid Z.A., Abu-Rmeileh N.M., Acosta-Cazares B., Acuin C., Adams R.J., Aekplakorn W., Afsana K., Aguilar-Salinas C.A. (2017). Worldwide trends in body-mass index, underweight, overweight, and obesity from 1975 to 2016: A pooled analysis of 2416 population-based measurement studies in 128·9 million children, adolescents, and adults. Lancet.

[7] Manyanga T., El-Sayed H., Doku D.T., Randall J.R. (2014). The prevalence of underweight, overweight, obesity and associated risk factors among school-going adolescents in seven African countries. BMC Public Health.

[8] Ghana Statistical Service (GSS)/Ghana Health Service (GHS)/ICF International (2015). Demographic and Health Survey 2014.

[9] Prince A.K., Laar A. (2014). Nutritional status of school-aged children in the Nkwanta South District-Volta Region of Ghana. Eur. Sci. J..

[10] Alicke M., Boakye-Appiah J.K., Abdul-Jalil I., Henze A., Van Der Giet M., Schulze M.B., Schweigert F.J., Mockenhaupt F.P., Bedu-Addo G., Danquah I. (2017). Adolescent health in rural Ghana: A cross-sectional study on the co-occurrence of infectious diseases, malnutrition and cardio-metabolic risk factors. PLoS ONE.

[11] Aryeetey R., Lartey A., Marquis G.S., Nti H., Colecraft E., Brown P. (2017). Prevalence and predictors of overweight and obesity among school-aged children in urban Ghana. BMC Obes..

[12] Afrifa–Anane E., Agyemang C., Nii S., Codjoe A., Ogedegbe G., Aikins A. (2015). The association of physical activity, body mass index and the blood pressure levels among urban poor youth in Accra, Ghana. BMC Public Health.

[13] Moubarac J., Martins A.P.B., Martins B., Claro R.M., Levy R.B., Cannon G., Monteiro C.A. (2012). Consumption of ultra-processed foods and likely impact on human health. Evidence from Canada. Public Health Nutr..

[14] Amos P.M., Intiful F.D., Boateng L. (2012). Factors that were found to influence Ghanaian adolescents’ eating habits. SAGE Open.

[15] Doku D., Koivusilta L., Raisamo S., Rimpelä A. (2011). Socio-economic differences in adolescents’ breakfast eating, fruit and vegetable consumption and physical activity in Ghana. Public Health Nutr..

[16] Anyaegbu E., Dharnidharka V. (2015). Hypertension in the teenager. Pediatr. Clin. N. Am..

[17] Aglony M., Acevedo M., Ambrosio G. (2009). Hypertension in adolescents. Expert Rev. Cardiovasc. Ther..

[18] WHO (2006). Adolescent Nutrition: A Review of the Situation in Selected South-East Asian Countries.

[19] Rutstein S.O., Rojas G., DHS (2006). Guide to DHS Statistics. Demographic and Health Surveys Methodology.

[20] De Onis M., Onyango A.W., Borghi E., Siyam A., Nishida C., Siekmann J. (2007). Development of a WHO growth reference for school-aged children and adolescents. Bull. World Health Organ..

[21] NIH (2005). The Fourth Report on the Diagnosis, Evaluation, and Treatment of High Blood Pressure in Children and Adolescents.

[22] Lassi Z.S., Moin A., Das J.K., Salam R.A., Bhutta Z.A. (2017). Systematic review on evidence-based adolescent nutrition interventions. Ann. N. Y. Acad. Sci..

[23] Amugsi D.A., Mittelmark M.B., Lartey A., Matanda D.J., Urke H.B. (2014). Influence of childcare practices on nutritional status of Ghanaian children: A regression analysis of the Ghana Demographic and Health Surveys. BMJ Open.

[24] WHO/UNICEF (2006). Core Questions on Drinking-Water and Sanitation for Household Surveys.

[25] Reddy V.B., Kusuma Y.S., Pandav C.S., Goswami A.K., Krishnan A. (2017). Water and sanitation hygiene practices for under-five children among households of sugali tribe of chittoor district, Andhra Pradesh, India. J. Environ. Public Health.

[26] Bryant K., Anhalt J., Dar B., Wiener C., Doehring J., Rosenfeld J. (2014). Establishing a baseline for water, sanitation and hygiene knowledge, attitudes, and practices in rural Ethiopia. J. Glob. Health.

[27] Owusu K., Waylen P. (2009). Trends in spatio-temporal variability in annual rainfall in Ghana (1951–2000). Weather.

[28] Barros A.J.D., Hirakata V.N. (2003). Alternatives for logistic regression in cross-sectional studies: An empirical comparison of models that directly estimate the prevalence ratio. BMC Med. Res. Methodol..

[29] Coutinho L.M.S., Scazufca M., Menezes P.R. (2008). Methods for estimating prevalence ratios in cross-sectional studies. Rev. Saude Publica.

[30] Berglund P.A. (2014). Analysis of Survey Data Using the SAS SURVEY Procedures: A Primer.

[31] Bosch A.M., Baqui A.H., van Ginneken J.K. (2008). Early-life determinants of stunted adolescent girls and boys in Matlab, Bangladesh. J. Health Popul. Nutr..

[32] Zazara D.E., Arck P.C. (2019). Developmental origin and sex-specific risk for infections and immune diseases later in life. Semin. Immunopathol..

[33] Doss C. (2013). Intrahousehold Bargaining and Resource Allocation in Developing Countries.

[34] Dapi L.N., Urban J., Nouedoui C., Stenlund H., Håglin L. (2009). Socioeconomic and gender differences in adolescents’ nutritional status in urban Cameroon, Africa. Nutr. Res..

[35] Gyamfi D., Obirikorang C., Acheampong E., Asamoah E.A., Sampong B.B., Batu E.N., Anto E.O. (2019). Weight management among school-aged children and adolescents: A quantitative assessment in a Ghanaian municipality. BMC Pediatr..

[36] Ecker O., Fang P., Covic N., Hendriks S.L. (2016). Economic development and nutrition transition in Ghana: Taking stock of food consumption patterns and trends. Achieving a Nutrition Revolution for Africa: The Road to Healthier Diets and Optimal Nutrition.

[37] Howard L.L. (2013). Food insecurity experiences predict children’s fruit and vegetable consumption in the USA. ISRN Nutr..

[38] Stevens B., Watt K., Brimbecombe J., Clough A., Judd J., Lindsay D. (2016). The role of seasonality on the diet and household food security of pregnant women living in rural Bangladesh: A cross-sectional study. Public Health Nutr..

[39] Satija A., Hu F.B. (2012). Cardiovascular benefits of dietary fiber. Curr. Atheroscler. Rep..

[40] Inge T.H., King W.C., Jenkins T.M., Courcoulas A.P., Mitsnefes M., Flum D.R., Wolfe M.B., Pomp A., Dakin G.F., Khandelwal S. (2018). The Effect of obesity in adolescence on adult health status. Pediatrics 2013.

[41] Van Abeelen A., De Rooij S., Osmond C., Painter R.C., Veenendaal M., Bossuyt P., Elias S., Grobbee D., Van Der Schouw Y.T., Barker D. (2011). The sex-specific effects of famine on the association between placental size and later hypertension. Placenta.

[42] Bundy D.A.P., Silva N., de Horton S., Jamison D.T., Patton G.C. (2017). Child and Adolescent Health and Development. Disease Control Priorities.

[43] Rotteveel J., Van Weissenbruch M.M., Twisk J.W.R., Waal H.A.D.-V.D. (2008). Infant and childhood growth patterns, insulin sensitivity, and blood pressure in prematurely born young adults. Pediatrics.

[44] Boah M., Azupogo F., Amporfro D.A., Abada L.A. (2019). The epidemiology of undernutrition and its determinants in children under five years in Ghana. PLoS ONE.

[45] Jee S.H., He J., Whelton P.K., Suh I., Klag M.J. (1999). The effect of chronic coffee drinking on blood pressure a meta-analysis of controlled clinical trials. Hypertension.

[46] Zimmerman F.J., Bell J.F. (2010). Associations of television content type and obesity in children. Am. J. Public Health.

[47] Darin-Mattsson A., Fors S., Kåreholt I. (2017). Different indicators of socioeconomic status and their relative importance as determinants of health in old age. Int. J. Equity Health.

[48] Cumming O., Cairncross S. (2016). Can water, sanitation and hygiene help eliminate stunting? Current evidence and policy implications. Matern. Child Nutr..

[49] Azupogo F., Abdul-Rahaman F., Gyanteh B., Atosona A. (2019). Hygiene and sanitation practices and the risk of morbidity among children 6–23 months of age in Kumbungu District, Ghana. Adv. Public Health.

[50] Riley A.P. (1994). Determinants of adolescent fertility and its consequences for maternal health, with special reference to rural Bangladesh. Ann. N. Y. Acad. Sci..

[51] WFP (2009). Comprehensive Food Security and Vulnerability Analysis (CFSVA). Republic of Ghana.

[52] Cooke E., Hague S., McKay A. (2016). The Ghana Poverty and Inequality Report—2016: Using the 6th Ghana Living Standards Survey.

[53] Bingenheimer J.B., Reed E. (2014). Risk for coerced sex among female youth in Ghana: Roles of family context, School enrollment and relationship experience. Int. Perspect. Sex. Reprod. Health.

[54] Senadza B. (2012). Education inequality in Ghana: Gender and spatial dimensions. J. Econ. Stud..

[55] Gbedemah C., Jones N., Pereznieto P. (2010). Gendered risks, poverty and vulnerability in Ghana: Is the LEAP cash transfer programme making a difference?. ODI Proj. Brief. Overseas Dev. Inst..

[56] Soekarjo D.D., Shulman S., Graciano F., Moench-Pfanner R. (2014). Improving Nutrition for Adolescent Girls in Asia and the Middle East: Innovations are Needed.

[57] Li J., Tsuprykov O., Yang X., Hocher B. (2016). Paternal programming of offspring cardiometabolic diseases in later life. J. Hypertens..

[58] Hawkes C., Ruel M.T., Salm L., Sinclair B., Branca F. (2020). Double-duty actions: Seizing programme and policy opportunities to address malnutrition in all its forms. Lancet.

[59] Wei D., Brigell R., Khadka A., Perales N., Fink G. (2019). Comprehensive school-based health programs to improve child and adolescent health: Evidence from Zambia. PLoS ONE.

