# Supplementary material for: Malnutrition, Hypertension Risk, and Correlates: An Analysis of the 2014 Ghana Demographic and Health Survey Data for 15–19 Years Adolescent Boys and Girls
Source: Nutrients. 2020 Sep 8;12(9):2737. doi: 10.3390/nu12092737 (PMC7551149; doi:10.3390/nu12092737)
Supplement: Supplementary file 1 [file nutrients-12-02737-s001.pdf]

Supplementary Data

Table S1: Decision making score for adolescent males and females in DHS 2014 data

Variables	Sex	
	Female	Male
**Index of attitude toward wife beating**		
Wife beating is justified if:		
1. Goes out without telling him	√	√
2. Neglects the children	√	√
3. Argues with him	√	√
4. Refuses to have sex with him	√	√
5. Burns the food	√	√
**The maximum attainable score for attitude toward wife beating**	**5**	**5**
Index of property ownership:		
1. Owns a house alone or jointly	√	√
2. Owns land alone or jointly	√	√
**The maximum attainable score for property ownership**	**2**	**2**
**Summated empowerment index**	**7**	**7**

Table S2: Scoring format from responses for decision-making indexes

**Index of attitude toward wife beating scoring format**	**Index of property ownership Scoring format**
No=1	Does not own=0
Yes =0	Alone only=1
	Jointly only=1
	Both alone and jointly=1

Table S3: Improved household water, hygiene and sanitation index (WASH)

No	Variable	Score
**1**	**Household water index (WI): score range 0-3**	
1.1	Improved source of drinking	
	▪ Piped water supply into the dwelling	1
	▪ Piped water to a yard/plot	
	▪ Public tap/standpipe	
	▪ Tube well/borehole	
	▪ Protected dug well	
	▪ Protected spring	
	▪ Rainwater	
	▪ If the source of drinking water is packaged/sachet-water or bottled and source of non-drinking water is improved	
	▪ Any other water source	0
1.2	Round-trip time in water haulage between household and water source	
	▪ If the time spent ≤ 30 minutes	1
	▪ If time spent > 30 minutes	0
1.3	Anything done to water to make it safe for drinking by household	
	▪ Any treatment (boiling, bleach/chlorine, filter, strain through cloth, camphor/naphthalene, solar disinfection, purification tablet, stand and settle or other treatment)	1
	▪ No treatment	0
**2**	**Household sanitation index (HSI): score range 0-2**	
2.1	Improved household sanitation facility using the type of toilet	
	▪ Flush/pour-flush toilet or latrine that flushes to a sewer, septic tank or pit.	1
	▪ Ventilated improved pit (VIP) latrine	
	▪ Pit latrines with the pit well covered by a slab	
	▪ Composting toilets are also considered improved	
	▪ Any other toilet	0
2.2	The household does not share toilet facility with others	1
**3**	**Household hygiene index (HHI): score range 0-3**	
3.1	If the household has a place for washing hands	1
3.2	Presence of water at a handwashing place in the household	1
3.3	Soap and other detergents available for hand washing in household	1
	**Maximum attainable score for household water, hygiene and sanitation index (WASH)**	**8**

Table S4. Multivariate Predictors of Height-For-Age Z-Score (HAZ) and Body-Mass-Index-For-Age Z-Score (BAZ): A Comparative Analysis for Adolescent Boys and Girls from the 2014 Ghana Demographic Health Survey (GDHS) Data

**Variables**	**HAZ**						**BAZ**					
	**Girls (n=857)**		**Boys (n=870)**		**Pooled (n=1727)**		**Girls (n=857)**		**Boys (n=870)**		**Pooled (n=1727)**	
	**Estimate (β)**	**S.E (β)**	**Estimate (β)**	**S.E (β)**	**Estimate (β)**	**S.E (β)**	**Estimate (β)**	**S.E (β)**	**Estimate (β)**	**S.E (β)**	**Estimate (β)**	**S.E (β)**
Sex												
Boy					-0.17	0.13					-0.27**	0.14
Girl					Ref.						Ref.	
Age	-0.08***	0.03										
Girl Menstruated in the last 6 weeks												
Yes	0.41***	0.13					0.25**	0.12				
No	Ref.						Ref.					
Adolescent has experienced first sex												
Yes	0.22***	0.07			0.14***	0.04	0.18**	0.07	0.34****	0.08	0.23****	0.05
No	Ref.				Ref.		Ref.					
Adolescent has a child												
Yes					-0.36***	0.13			0.51**	0.26		
No (Ref.)					Ref.							
**Interaction term sex* adolescent has a child**												
Girl *Has a child					-0.39**	0.16						
Highest educational level of girl												
No education	-0.38**	0.16	0.29	0.22	-0.10	0.13						
Primary school	-0.21**	0.09	-0.35****	0.10	-0.26****	0.06						
Secondary education /Higher	Ref.		Ref.		Ref.							
Ethnicity												
Akan					-0.14**	0.06						
Mole-Dagbani					0.05	0.07						
Other					Ref.							
Marital status												
Currently married							-0.69**	0.30				

Never married							Ref.					
Empowerment score					0.06**	0.03					0.07**	0.03
**An interaction term for sex*empowerment score**												
Girl * empowerment score											0.09**	0.03
Water, hygiene and sanitation score			0.06**	0.02								
Household wealth index												
Poorest	-0.21*	0.11	-0.29**	0.12	-0.16	0.10	-0.42****	0.11	-0.21	0.13	-0.33****	0.08
Poorer	-0.27***	0.11	-0.43****	0.13	-0.27***	0.10	-0.44****	0.11	-0.37***	0.13	-0.43****	0.09
Middle	-0.24**	0.12	-0.30**	0.13	-0.23**	0.11	-0.15	0.12	-0.29**	0.14	-0.22**	0.09
Richer	-0.08	0.12	-0.16	0.12	-0.10	0.12	-0.16	0.14	-0.02	0.14	-0.09	0.09
Richest	Ref.		Ref.		Ref.		Ref.		Ref.		Ref.	
**An interaction term for Sex * Household Wealth Quintile**												
Boy* Poorest					-0.35**	0.15						
Boy*Poorer					-0.33**	0.16						
Boy*Middle					-0.21	0.18						
Boy*Richer					-0.15	0.17						
Boy*Richest					Ref.							
Agro-ecological zone												
Coastal savannah	-0.09	0.08										
Guinea/Sudan savannah	0.19*	0.10										
Forest	Ref.											
**Model Fit Statistics**												
R-Square	0.07		0.09		0.11		0.07		0.07		0.17	
Root MSE	0.85		0.88		0.87		0.85		0.78		0.82	
Model (F-Value)	3.80****		7.49***		10.51****		6.01****		5.63***		29.38***	

Ref, Reference group; *P≤ 0.1; ** P≤0.05; ***P≤0.01; **** P≤0.001

Table S5. Multivariate Predictors of Blood Pressure: A Comparative Analysis for Adolescent Boys and Girls from the 2014 Ghana Demographic Health Survey (GDHS) Data

**Variables**	**Systolic blood pressure**						**Diastolic blood pressure**					
	**Girls (n=857)**		**Boys (n=870)**		**Pooled (n=1727)**		**Girls (n=857)**		**Boys (n=870)**		**Pooled (n=1727)**	
	**Estimate (β)**	**S.E (β)**	**Estimate (β)**	**S.E (β)**	**Estimate (β)**	**S.E (β)**	**Estimate (β)**	**S.E (β)**	**Estimate (β)**	**S.E (β)**	**Estimate (β)**	**S.E (β)**
Sex												
Boy					3.35**	1.49					-1.56***	0.49
Girl					Ref.						Ref.	
Age			1.37****	0.34	0.74****	0.21			0.32*	0.17	0.40**	0.17
Adolescent has experienced first sex												
Yes	-2.34**	1.09	2.89***	1.12	-0.46	0.85						
No	Ref.				Ref.							
**Interaction term sex* experienced first sex**												
Girl*experienced first sex					3.84***	1.26						
Adolescent has a child												
Yes			18.22****	1.61							-2.47**	1.10
No												
Adolescent exercised in the past 10 minutes												
Yes	4.08***	1.47										
No	Ref.				Ref.							
Ate in the past 10 minutes												
Yes			2.55****	0.81	1.69***	0.61						
No			Ref.		Ref.							
Had coffee in the past 10 minutes												
Yes			6.39****	1.23	-2.49	2.22					-1.01	1.49
No			Ref.		Ref.						Ref.	
**An interaction term for sex*had coffee in the past 10 minutes**												
Boy * had coffee in the past 10					8.32***	2.61					7.32****	2.15

minutes												
Frequency of fruit intake in the past week			0.32*	0.17								
Visited a health facility in the last 6 months												
Yes					-2.25***	0.79						
No					Ref.							
Marital status												
Currently married			-21.70****	5.88					-4.44***	1.67		
Formerly married			N/A						N/A			
Never married			Ref.									
Household owns land usage for agriculture												
Yes							-1.44**	0.69				
No							Ref.					
Household wealth index												
Poorest			5.84****	1.40	1.06	1.48						
Poorer			1.45	1.41	-0.88	1.50						
Middle			1.92	1.27	1.00	1.51						
Richer			1.63	1.27	0.28	1.48						
Richest			Ref.		Ref.							
**An interaction term for Sex * Household Wealth Quintile**												
Boy* Poorest					3.91**	1.75						
Boy*Poorer					2.25	1.93						
Boy*Middle					1.14	1.88						
Boy*Richer					1.40	1.88						
Boy*Richest					Ref.							
Agro-ecological zone												
Coastal savannah			1.90**	0.93	1.07	0.69					-0.52	0.55
Guinea/Sudan savannah			-3.48**	1.53	-2.68***	0.93					-2.11****	0.63
Forest			Ref.		Ref.						Ref.	
BAZ category												
Underweight	-6.90	5.19	-2.53	1.29	-3.67**	1.88			-2.98**	1.51	-2.68*	1.53
Overweight	2.98***	1.02	4.71****	1.39	3.14****	0.97			1.98***	0.76	1.35*	0.78

Normal weight	Ref.		Ref.		Ref.				Ref.		Ref.	
Adolescent is stunted												
Yes			-3.86***	1.25	-3.23***	1.09						
No			Ref.		Ref.							
Agro-ecological zone												
Coastal savannah											-0.48	0.55
Guinea/Sudan savannah											-1.97****	0.61
Forest											Ref.	
**Model Fit Statistics**												
R-Square	0.04		0.15		0.15		0.01		0.02		0.03	
Root MSE	9.99		10.46		10.30		8.01		8.05		8.01	
Model (F-Value)	5.83****		35.99****		16.76****		4.39**		3.58****		5.32****	

Ref, Reference group; *P≤ 0.1; ** P≤0.05; ***P≤0.01; **** P≤0.001; BAZ, Body-mass index-for-age Z-score; N/A, not applicable because none of the Boy adolescents was formerly married; N/A, none of the adolescent boys was formerly married
